# A pleiotropic interaction between vision loss and hypermelanism in *Astyanax mexicanus* cave x surface hybrids

**DOI:** 10.1186/s12862-016-0716-y

**Published:** 2016-06-30

**Authors:** Joshua B. Gross, Amanda K. Powers, Erin M. Davis, Shane A. Kaplan

**Affiliations:** Department of Biological Sciences, University of Cincinnati, Cincinnati, Ohio 45223 USA; Department of Biological Sciences, University of Cincinnati, Rieveschl Hall Room 711B, 312 Clifton Court, Cincinnati, Ohio 45221 USA

**Keywords:** Regressive evolution, Trait loss, Quantitative trait locus analysis, Phenotypic effects

## Abstract

**Background:**

Cave-dwelling animals evolve various traits as a consequence of life in darkness. Constructive traits (e.g., enhanced non-visual sensory systems) presumably arise under strong selective pressures. The mechanism(s) driving regression of features, however, are not well understood. Quantitative trait locus (QTL) analyses in *Astyanax mexicanus* Pachón cave x surface hybrids revealed phenotypic effects associated with vision and pigmentation loss. Vision QTL were uniformly associated with reductions in the homozygous cave condition, however pigmentation QTL demonstrated mixed phenotypic effects. This implied pigmentation might be lost through both selective and neutral forces. Alternatively, in this report, we examined if a pleiotropic interaction may exist between vision and pigmentation since vision loss has been shown to result in darker skin in other fish and amphibian model systems.

**Results:**

We discovered that certain members of Pachón x surface pedigrees are significantly darker than surface-dwelling fish. All of these “hypermelanic” individuals demonstrated severe visual system malformations suggesting they may be blind. A vision-mediated behavioral assay revealed that these fish, in stark contrast to surface fish, behaved the same as blind cavefish. Further, hypermelanic melanophores were larger and more dendritic in morphology compared to surface fish melanophores. However, hypermelanic melanophores responded normally to melanin-concentrating hormone suggesting darkening stemmed from vision loss, rather than a defect in pigment cell function. Finally, a number of genomic regions were coordinately associated with both reduced vision and increased pigmentation.

**Conclusions:**

This work suggests hypermelanism in hybrid *Astyanax* results from blindness. This finding provides an alternative explanation for phenotypic effect studies of pigmentation QTL as stemming (at least in part) from environmental, rather than exclusively genetic, interactions between two regressive phenotypes. Further, this analysis reveals persistence of background adaptation in *Astyanax.* As the eye was lost in cave-dwelling forms, enhanced pigmentation resulted. Given the extreme cave environment, which is often devoid of nutrition, enhanced pigmentation may impose an energetic cost. Such an energetic cost would be selected against, as a means of energy conservation. Thus, the pleiotropic interaction between vision loss and pigmentation may reveal an additional selective pressure favoring the loss of pigmentation in cave-dwelling animals.

**Electronic supplementary material:**

The online version of this article (doi:10.1186/s12862-016-0716-y) contains supplementary material, which is available to authorized users.

## Background

The constant darkness and reduced nutrition of subterranean environments drives convergent phenotypic changes across distantly related cave animals [1]. Irrespective of significant phylogenetic and geographic distances, ‘constructive’ phenotypes are augmented in the darkness of the cave (e.g., expansion of non-visual sensation; [2]), while ‘regressive’ phenotypes are diminished or lost [3]. Constructive phenotypes are generally regarded as evolving under selective pressure [4], however the precise evolutionary mechanism(s) driving trait regression remain incompletely understood [5].

Two conspicuous phenotypes diminished or lost in cave animals are vision [6] and pigmentation [7, 8]. Classical genetic crosses of cave- and surface-dwelling forms of the freshwater characiform *Astyanax mexicanus* indicated that these traits are mediated by numerous genetic loci [9]. To understand the evolutionary mechanisms governing trait loss, Protas et al. [10] performed a quantitative trait locus (QTL) analysis of eye size and pigmentation in an experimental F_2_ pedigree of >500 hybrid individuals [10]. Phenotypic effect analyses revealed that cave alleles at *every* eye QTL were associated with reductions in eye size [10]. However, the polarities of pigmentation QTL (i.e., melanophore numbers) were mixed. In some cases, the homozygous cave condition was associated with a decrease in the number of melanophores, while in other cases the homozygous cave condition was associated with *more* melanophores [10].

Different phenotypic effects of pigmentation and vision loci may indicate that trait regression evolves through different mechanisms. For instance, the uniform reduction of eye size associated with cave alleles is *consistent* with selection against an eye in the darkness of a cave [10]. Contrarily, diverse polarities associated with pigmentation loss may indicate pigment cell numbers change as a consequence of diverse forces, including selection and genetic drift [10].

In principle, however, an alternative (or additional) explanation for this phenomenon may be an *interaction* between vision loss and pigmentation. Such an interaction might be predicted in hybrid individuals inheriting certain allelic combinations associated with vision and pigmentation variation. For instance, interactions between Mendelian pigmentation phenotypes have been previously documented in hybrid *Astyanax* offspring [11]. Individuals inheriting two copies of the surface-dwelling (intact) form of *Mc1r*, but two copies of the Pachón (non-functional) form of *Oca2*, are phenotypically albino owing to a non-functional Oca2 protein channel [12].

Although interactions *between* pigmentation and vision could be similarly predicted, none have been characterized in hybrid individuals. This may be due to the fact that vision and pigmentation in *Astyanax* are complex phenotypes mediated by several regions of the genome [10, 13, 14]. Therefore, deciphering complex interactions between multiple loci would be challenging. Interestingly, however, at the gross phenotypic level, Protas et al. [10] noted three melanophore phenotypes that were significantly *negatively* correlated with eye size.

Additional supporting evidence for a potential interaction between vision and pigmentation comes from prior analyses carried out in *eyeless* axolotls, blinded *Xenopus*, and *lakritz* zebrafish mutants. These studies revealed that darker body pigmentation occurs in the absence of visual system input [15–17]. We sought to determine if a similar relationship exists between vision and pigmentation in *Astyanax* hybrids. *Astyanax mexicanus* comprises both cave- and surface-dwelling morphs that can be bred to produce viable hybrid offspring [18]. Early classical genetic studies suggested Mendelian pigmentation traits and eye size were unlinked [19]; however, a contemporary genetic analysis revealed several ‘melanophore number variation’ and ‘eye size’ QTL reside near one another [10].

Here, we investigated if an interaction between reduced vision and increased pigmentation may occur in certain hybrid *Astyanax* fish. Interestingly, Protas et al. [10] reported a significant inverse relationship between pigmentation and eye size in their QTL analyses of eye reduction and three melanophore number traits: MelD (−0.170), MelA (−0.184), and MelL (−0.231; supplementary information in [10]). The fourth melanophore trait scored in their study (MelE) demonstrated a negative, but insignificant, correlation with eye size (−0.050). We reanalyzed the same experimental pedigree from Protas et al. [10] and discovered a small number of individuals (*n* = 11) with very dark pigmentation (>95th percentile) that harbored some of the smallest eye size measures in the entire pedigree (<3rd percentile) [10]. We bred a second experimental F_2_ pedigree that also revealed a few hybrid individuals with melanic pigmentation significantly exceeding the darkness of wild type surface-dwelling parents. Using quantitative and qualitative scoring strategies, we identified ten ‘hypermelanic’ individuals from this F_2_ pedigree (*n* = 129) with pigmentation significantly darker than surface fish reared under a normal 12 h light:dark cycle. Interestingly, every hypermelanic individual demonstrated structural abnormalities associated with the eye, including substantial reduction in overall eye size.

Taken together, this work implies an interaction exists between vision loss and dark pigmentation in *Astyanax* cave x surface hybrids, and the polarity of pigmentation and vision QTL may be partially influenced by the indirect interaction between these two regressive phenotypes. Further, this analysis suggests that as the eye was lost early in cave-dwelling lineages, enhanced pigmentation may have resulted, imposing an energetic cost in the nutrient-poor environment of the cave. This energetic cost was likely selected against, as a means of conserving energy, and contributing to the regression of pigmentation. Thus, an interaction between vision and pigmentation reveals a potential *negative* selective force, which may contribute to pigmentation loss in cave-dwelling organisms.

## Methods

### Breeding and animal husbandry

Animals were maintained under identical rearing conditions in a satellite fish husbandry room at the University of Cincinnati. Under typical rearing conditions, fish are housed under a 12 h light:12 h dark photic schedule and fed dry flake food (TetraMin Pro), administered once daily. All fish were maintained in a custom husbandry unit (Aquaneering, San Diego, CA) receiving reverse-osmosis water from a central facility and maintained at a pH of 7.4 (±0.2) and conductivity of 800 μS (±50 μS). To avoid cross-contamination and optimize health of the fish, each tank was maintained with separate water delivery and drainage. A recirculating pump was used to provide filtration through coarse and fine mechanical filters, a biofilter, a micron filter, and a UV filter. All individuals were housed in 5- or 10-gallon glass, group rearing tanks, or individually in 1-liter BPA-free plastic tanks. The only exception to these conditions were certain surface individuals that were reared under a 12 h dark:12 h dark cycle in “black out” tanks to evaluate the robustness of their background adaptation. These tanks were lined on the outside with black construction paper to eliminate light introduction to the tank (with the exception of a 2 cm hole in the top of the tank to permit water flow).

In this study, we analyzed a Pachón x surface fish experimental F_2_ cross (“Asty12”) previously published by Protas et al. [10] (see below). A second experimental F_2_ pedigree (“Asty66”) was maintained in our lab and also analyzed in this study. The members of the “Asty66” experimental pedigree were full-siblings, bred from a male (“Asty17-02”) and female (“Asty03-11”) Pachón x surface F_1_ hybrid crossed in the Borowsky laboratory (New York University). We also analyzed Pachón cavefish (“Asty138” pedigree) whose parents were originally collected in the wild from the Pachón cave (Sierra de El Abra region, Mexico) around 2001; and, surface-dwelling *Astyanax* individuals (“Asty155” and “Asty152”) derived from individuals collected at Arroyo Sarco in the Río Sabinas and the Río Valles drainages near Ciudad Valles, Mexico. All of these individuals were collected and generously provided to us by Dr. Richard Borowsky (New York University).

This study was performed in accordance with the Guide for the Care and Use of Laboratory Animals of the National Institutes of Health. The Institutional Animal Care and Use Committee (IACUC) of the University of Cincinnati approved our Animal Use Protocol (Number 10-01-21-01). All fin clips and scale removal involved the use of anesthesia (a buffered solution of tricaine methylsulphonate; MS-222) to minimize or eliminate suffering.

### Phenotypic analyses

#### Eye Measurements

Fish were imaged in profile on both sides of the face at 7.81× under identical illumination and bright field conditions using a Leica stereomicroscope (M205FA, Leica Application Suite v3.8). Eye size was quantified using the ‘polygon selection’ and ‘measurement’ tools in ImageJ (NIH, Bethesda, Maryland) for three metrics: 1) pupil area (px^2^), 2) eyeball area (px^2^) and 3) orbit area (px^2^). Pixel area (px^2^) was converted to millimeter area (mm^2^) using scale bar measurements and Microsoft Excel (Mac 2011 v14.5.5). Area measurements were compared across hypermelanic and surface L/D fish using a Student’s T-test (JMP 11).

#### Melanophore scoring

Melanosome aggregation assays evaluated melanophores present on scales isolated from the dorsal flank, just anterior to the dorsal fin. Single scales were isolated from the dorsal flank of hypermelanic (“Asty66”; *n* = 10 individuals) and surface fish (“Asty 155 and 152”; *n* = 12 individuals) by gentle removal with the #5 fine forceps (World Precision Instruments, Sarasota, FL). Scales were immediately placed in 1 mL of sterile 1× Danieau’s solution (Cold Springs Harbor Protocols) in a 48-well plate. Each scale was imaged using montage imaging under identical bright field conditions using a Leica stereomicroscope (M205FA) at 80× magnification (brightness = 60 %, saturatio*n* = 1.25, automatic exposure; Leica Application Suite v3.8). Each scale was then immersed in 1 μM of melanin concentrating hormone (“MCH”; Sigma-Aldrich) and re-imaged following 5 min of treatment. Control scales were treated with 1× Danieau’s solution. Melanophore areas were measured from pre- and post-treatment images using the polygon selection and measurement tools in ImageJ. Three melanophores were measured on each scale for pre- and post-treatment with MCH. Additionally, we measured relative darkness (white = 0, black = 255) of each melanophore using the “mean” function in ImageJ, as previously described [12]. Surface and hypermelanic scales were compared using a Student’s T-test (StatPlus v5.8).

### Visual system scoring

We developed a “half-moon” assay as a proxy for visual function in surface fish, hypermelanic hybrids, and eyeless Pachón cavefish. The half-moon arena was created using a cylindrical Pyrex® bowl (190 × 100 mm^3^) separated into equal sized semi-circles, and covered with black and white contact paper. This yielded a three-dimensional circular arena in which half of the area had a black background, and the other half had a white background.

We mounted a digital camera (Nikon Coolpix S8000) directly above the arena to record fish behavior. All trials were carried out under identical, ambient illumination conditions. Each fish was randomly assigned a trial, which began with a 1-min acclimation period prior to the 1-min trial recording. Between each trial, the arena was completely cleaned and filled with fresh system water. Fish were scored for the number of midline crosses (collected from video data using manual thumb-clickers) and the amount of time (seconds) spent in the white and black sections of the arena. Each fish completed three trials, from which their scores were averaged. Each group was compared using an ANOVA across all scoring metrics (JMP 11).

### Digital coloration and greyscale analyses

To quantify and compare coloration across different individuals, we employed a technique described by Gross and Wilkens [20]. Briefly, fin clips processed for genomic DNA using Qiagen columns provided a measure of coloration. All fin clips were collected under anesthesia (see *Breeding and Animal Husbandry*) from the dorsal portion of the caudal fin, and processed in 180 μl of ATL Buffer (Qiagen) + 20 μl of proteinase K (10 mg/ml) solution. Following extraction and purification, the filter was carefully removed from the QiaQuick spin column and imaged under high-resolution magnification. Membrane-bound filtrate includes the melanin isolated from fin tissue, providing a measure of the coloration difference between individuals. All filters were imaged at 13.7× magnification under identical microscopy and lighting conditions.

All images were analyzed for digital coloration by overlaying a circular grid with 13 concentric and equally-spaced dots, each of which were sampled and scored using Adobe Photoshop software (PS3). Using the “eye dropper” tool, we recorded the relative contributions of each of the four CMYK channels (ranging from 0 to 100 %), and averaged these values across all 13 data points to generate a mean coloration value for each individual. We collected and analyzed fin clips from Pachón cavefish (*n* = 7), Surface L/D-reared (*n* = 13), Surface D/D-reared (*n* = 10), hypermelanic individuals (*n* = 10).

**Fig. 1:**
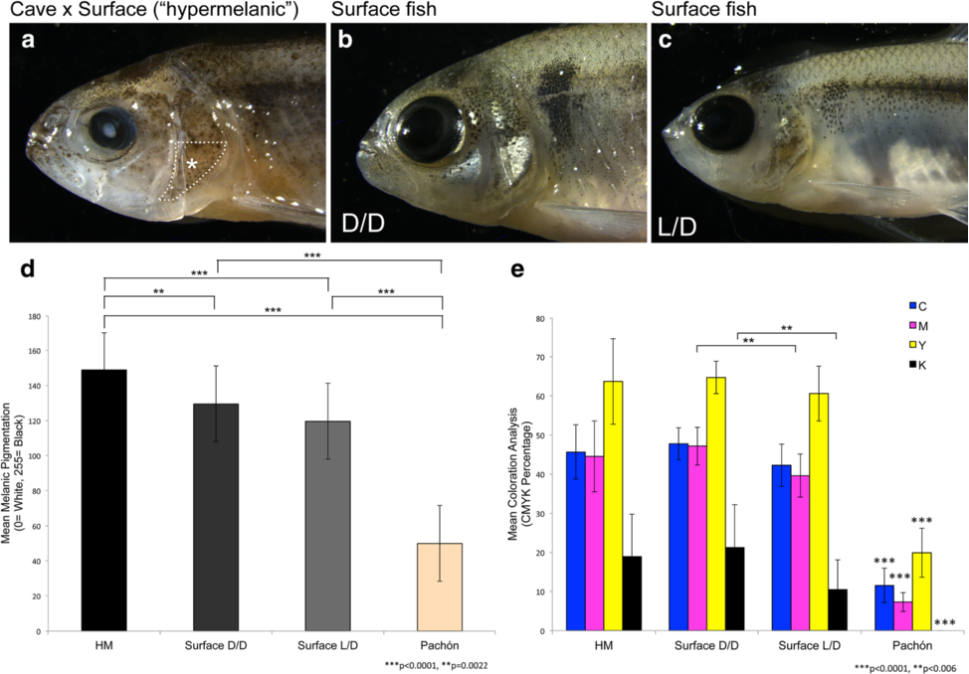
Hypermelanic cave x surface hybrid fish are significantly darker than wild type surface-dwelling fish. A representative hypermelanic individual from a cave x surface F_2_ pedigree (**a**), a DD surface fish maintained under a 24 h dark cycle (**b**) and a LD surface fish reared under a normal 12 h light: 12 h dark photic cycle (**c**) were imaged to illustrate differences in melanophore numbers and morphology (7.8× magnification). Mean pigmentation darkness (**d**) was measured from extracted melanin isolated from fin tissue via a greyscale analysis in ImageJ (0 = White, 255 = Black). Hypermelanic fish were significantly darker than DD and LD surface fish, as well as albino Pachón cavefish (**p = 0.0022; ***p < 0.0001; Bonferroni corrected p-value for significance = 0.0083). A mean coloration analysis (**e**) of the extracted fin tissue in Adobe Photoshop revealed no significant differences between hypermelanic individuals and DD reared surface fish across cyan, magenta, yellow and keyed (black) color channels, however, there were significant differences noted across color channels when comparing hypermelanic and surface DD fish to both surface LD and Pachón cavefish (**p = 0.006; ***p < 0.0001; Bonferroni corrected p-value for significance = 0.0083). Comparing hypermelanic fish (HM), Surface DD and Surface LD, significant differences in greyscale were noted for the following: HM ≠ DD, LD, and Pachón; DD and LD ≠ Pachón (**d**). Comparing all groups, significant differences in coloration were noted for the following color channels: Magenta: DD ≠ LD; Keyed: DD ≠ LD. Note that Pachón cavefish differed from all groups in all four coloration channels (**e**)

We also performed parallel measures of melanic darkness on each filter using ImageJ software as described in Gross et al. [12]. Briefly, each image was rendered in greyscale to discard color information and inverted before analyzing. This provided a quantifiable measure of darkness ranging in value from 0 to 255 (0 = white; 255 = black). Each image was loaded into ImageJ and the mean darkness value was collected in triplicate and averaged. As above, all mean values were collected for each group. All statistical analyses were carried out using JMP software (v11). To control for Type I error, we performed a Bonferroni correction for our greyscale and coloration analyses. We compared pair-wise coloration across four groups (hypermelanic, surface fish reared in light/dark conditions, surface fish reared in dark/dark conditions, and Pachón cavefish), and therefore tested six pairwise hypotheses. We set our α at 0.05, and therefore our Bonferroni correction tested each individual hypothesis for significance at α = 0.05/6 = 0.0083 (Fig. 1).

### Reanalyses of Protas et al. (2007)

We reevaluated data published by Protas et al. [10] to determine whether “hypermelanic” individuals may be present in their surface x Pachón cavefish F_2_ pedigree. We began by rank-ordering all individuals in this pedigree, based on phenotypic scores provided by the authors, to determine the percentile (“%ile”) ranking of each individual for each vision and pigmentation trait. The pigmentation traits included the following: “MelA” (melanophore count above the anal fin), “MelL” (melanophore count below the midlateral stripe), “MelD” (melanophore count below the dorsal fin), and “MelE” (melanophore count above the eye; [10]). Of the 539 experimental F_2_ individuals comprising this pedigree, 535 individuals were scored for eye size, 115 were scored for lens size, 128 individuals were scored for the MelA and MelL pigmentation traits, and 174 individuals were scored for both the MelD and MelE pigmentation traits. Four individuals were scored for all four melanophore traits; however, the majority of individuals were scored for only MelA and MelL, or only MelD and MelE.

We also examined the genotypes for each individual with simultaneously high values for pigmentation and low values for eye size (Table 1). These genotypes were drawn from Supplementary Document S3 (“*Astyanax* Genotypes and Phenotypes” in [10]). The loci we evaluated were the nearest markers to the peak LOD scores reported for the 18 pigmentation loci and 14 visual system loci (eye size = 8; lens size = 6; Additional file 1: Table S1). Finally, we reanalyzed the QTL data to identify loci near genomic regions associated with high melanophore numbers and low eye and lens size values in the homozygous cave condition. Genotype and phenotype values were reanalyzed to create phenotypic effect plots for each locus using r/QTL, as previously described [21]. Spearman rank-order correlations were performed using Microsoft Excel v.14.4.0 using the RealStatistics resource pack.

**Table 1:** Experimental F_2_ individuals demonstrating dark pigmentation and small eyes (data reanalyzed from Protas et al. 2007)

Pedigree Individual	Pigmentation Trait	Pigmentation Score	Pigmentation Rank (Percentile)	Eye Size Score	Eye Size Rank (Percentile)
397	MelLat	52	1/128 (100 %ile)	0.49948	8/535 (1.5 %ile)
390	MelLat	42	4/128 (97.7 %ile)	0.49627	7/535 (1.3 %ile)
375	MelLat	35	11/128 (92.2 %ile)	0.48033	6/535 (1.1 %ile)
383	MelAnal	38	14/128 (89.5 %ile)	0.46687	4/535 (0.75 %ile)
312	MelAnal	38	14/128 (89.5 %ile)	0.55552	20/535 (3.7 %ile)
375	MelAnal	26	28/128 (78.9 %ile)	0.48033	6/535 (1.1 %ile)
145	MelDorsal	35	16/174 (90.2 %ile)	0.5837	29/535 (5.4 %ile)
98	MelDorsal	35	14/174 (91.4 %ile)	0.58453	30/535 (5.6 %ile)
190	MelDorsal	51	2/174 (99.4 %ile)	0.60971	38/535 (7.1 %ile)
169	MelEyes	72.762	7/174 (96.6 %ile)	0.54895	18/535 (3.4 %ile)
98	MelEyes	77.444	3/174 (98.9 %ile)	0.58453	30/535 (5.6 %ile)
190	MelEyes	74.802	6/174 (97.1 %ile)	0.60971	38/535 (7.1 %ile)

This data was collected and analyzed from Supplementary Data Set 3 in Protas et al. (2007)

## Results

### Certain surface x cavefish F_2_ hybrids demonstrate dark melanic pigmentation combined with substantially reduced eye sizes

If a relationship exists between vision loss and pigmentation, we predicted that certain experimental F_2_ individuals would demonstrate high scores for pigmentation and low scores for eye size. To determine if this relationship exists, we reanalyzed phenotypic data reported in Protas et al. [10] (see Methods). Our analyses revealed a few individuals that simultaneously scored very high ‘melanophore number’ values (across all four phenotypes) with very small ‘eye size’ values (Table 1). For instance, three individuals (397, 390, 375) scored in the 100 %ile – 92.2 %ile for the MelL trait (1st, 4th, and 11th, respectively, across the entire pedigree) and collectively harbored eye size measurements in the bottom 1.5 %ile of the pedigree (Table 1). Similarly, three individuals (383, 312, 375) scored among the highest ranks for the MelA trait (14th, 14th, and 28th) and harbored eye size measurements in the bottom 3.7 %ile of the pedigree (Table 1). For the MelD trait, three individuals (145, 98, 190) harbored pigmentation scores ranging from the ~90–99 %ile, and eye size scores in the ~5–7 %ile (Table 1). Finally, three individuals (169, 98, 190) that scored in the ~96–99 %ile for MelE harbored eye size values in the bottom ~3–7 %ile (Table 1). Note that two of the same individuals, 375 and 98, ranked highly for the MelL/MelA scores, and MelD/MelE scores, respectively (Table 1). We also performed a set of Spearman rank-order correlation studies evaluating individuals in the bottom 10^th^ percentile of eye sizes (i.e., the individuals with the smallest eyes) and compared them against the entire pedigree. We discovered that the negative correlation between eye size and hyperpigmentation increased when assessing the individuals with the smallest eye sizes in the pedigree.

For example, The Spearman correlation coefficient for eye size and MelE was −0.067 across the entire pedigree, but was −0.235 for individuals in the bottom 10^th^ percentile of eye size; the correlation coefficient for eye size and MelA was −0.275 across the entire pedigree, and −0.532 for individuals in the bottom 10^th^ percentile of eye size; and the correlation coefficient for eye size and MelL was −0.208 across the entire pedigree, and was −0.522 for individuals in the bottom 10^th^ percentile of eye size. Interestingly, the Spearman correlation coefficient for eye size and MelD was −0.151 across the entire pedigree, but was −0.066 for individuals in the bottom 10^th^ percentile of eye size. Thus, the strength of the negative relationship in the smallest-eyed individuals diminished when evaluating the melanophores present on the *dorsal* region of the body. This may reflect a depletion (or migration) of melanophores from the dorsal flank, given that presence of ventral melanophores are unique to hypermelanic individuals. In any case, we did observe certain individuals demonstrating very small eye sizes alongside high scores for MelD (Table 1). Thus, certain individuals present in the pedigree evaluated by Protas et al. [10] clearly demonstrated dark melanic pigmentation combined with reduced eye size measures.

### Hypermelanism is present across different hybrid pedigrees

Our reanalysis revealed, in principle, a pleiotropic relationship may exist between vision and pigmentation loss in certain hybrid *Astyanax*. However, scores for surface-dwelling fish were not provided in the original report [10] to enable direct comparison between normal pigmentation in surface fish and “hypermelanic” fish. Furthermore, it was unclear whether individuals with darker pigmentation were unique to the pedigree analyzed by Protas et al. [10], or if such individuals could be observed in other pedigrees. To compare pigmentation levels between “hypermelanic” fish and surface dwelling fish, determine the potential recurrence of this phenomenon in a different pedigree, and directly evaluate pigmentation and eye size across individuals, we analyzed a second experimental F_2_ pedigree derived from a Pachón cave x surface fish cross.

We qualitatively assessed 129 individuals, from which we identified 10 fish demonstrating melanism that appeared to exceed that of wild type surface-dwelling fish. We quantified this pigmentation using a digital greyscale analysis of melanic pigmentation derived from tail fin-clip tissue (see [20]). When we compared melanin content isolated from hypermelanic, surface fish, and Pachón cavefish individuals, we found that hypermelanic fish were significantly darker than both morphotypes (*p* < 0.01; Fig. 1d). The average greyscale value for hypermelanic fish was 148.80, while the same value for surface fish was 119.62, and 49.90 in Pachón cavefish (255 = black; 0 = white). We then performed a digital coloration analysis in which each of four channels of the CMYK color mode were sampled and compared across Pachón cavefish, surface fish, and hypermelanic hybrid fish. All melanic groups were significantly different from Pachón cavefish (*p* < 0.0001; Fig. 1e). Surface DD fish demonstrated significantly higher levels of the keyed channel (“K”) and the magenta channel (“M”) compared to Surface LD fish – which reports the influence of black and reddish tones on digital coloration, respectively (*p* < 0.006; Fig. 1e). Pachón cavefish demonstrated negligible levels of “K” owing to the fact that they are albino (Fig. 1e) [20]. In sum, hypermelanic fish and dark-reared surface fish are quantifiably darker than surface fish reared under a typical 12 h light:12 h dark photic schedule.

### Four genomic regions are associated with both smaller eye size and greater numbers of melanophores

Eye size QTL polarities uniformly result in smaller eyes in the homozygous cave condition [10]. In our reanalysis, we identified several hybrid individuals with small eye size scores who exhibited hypermelanism (Table 1). So, we then decided to explore a potential genetic relationship in which the same locus, or closely linked loci, may be associated with reduced eye size *and* increased melanophores.

**Fig. 2:**
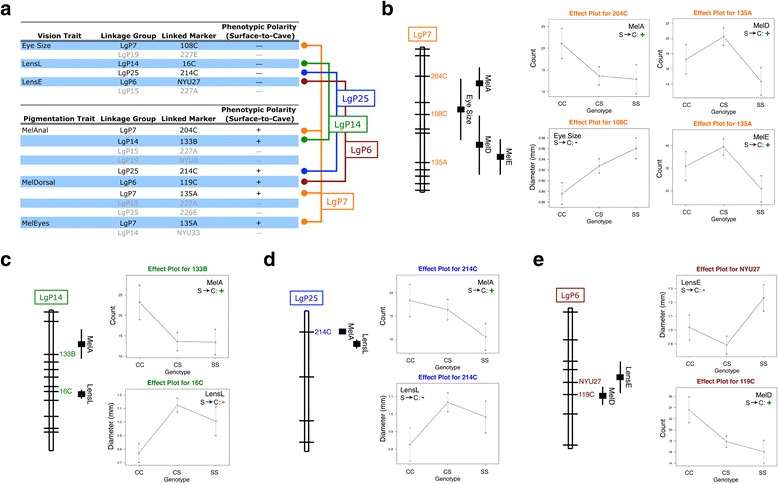
Reanalyses of Protas et al. (2007) revealed several regions of the genome are associated with opposite phenotypic effects for eye size and melanophore number. Four genomic regions localized to Pachón-based linkage groups (“LGP”) 6, 7, 14 and 25 harbor opposite phenotypic effects for vision and pigmentation (black font; **a**). QTL indicated in grey represent loci with congruent phenotypic effects (i.e. reductions in vision and pigmentation; (**a**). Three markers on LGP7 (204C, 108C, 135A) associated with one eye size and four pigmentation traits (MelA, MelD, MelE) demonstrate opposite phenotypic effects (**b**). Two markers on LGP14 (133B, 16C) associated with one lens size (LensL) and one pigmentation trait (MelA) demonstrate opposite phenotypic effects (**c**). One marker on LGP25 (214C) associated with one lens size (LensL) and one pigmentation trait (MelA) demonstrate opposite phenotypic effects (**d**). Two markers on LGP6 (NYU27, 119C) associated with one lens size (LensE) and one pigmentation trait (MelD) demonstrate opposite phenotypic effects (**e**). Data reanalyzed from Protas et al. [10]

We re-analyzed the data from Protas et al. [10] and identified six ‘eye size’ QTL (for three scored phenotypes) which mapped near ten melanophore number QTL (for three scored phenotypes; Fig. 2). If the absence of vision in certain members of the F_2_ generation led to darker pigmentation, we would anticipate that the homozygous cave genotype for certain eye size QTL would be associated with increased melanophore numbers. Indeed, we discovered four eye loss QTL mapped near six QTL associated with more melanophores in F_2_ individuals (Fig. 2). We note, however, that two ‘eye size’ QTL (on linkage groups 15 and 19) demonstrated similar phenotypic effects as melanophore numbers – i.e., the homozygous cave genotype was associated with fewer melanophores (Fig. 2a; grey font).

One eye size QTL associated with marker 108C on linkage group 7 overlapped with three melanophore QTL (linked to markers 135A and 204C) that demonstrate opposing phenotypic effects (Fig. 2b). The lens QTL on linkage group 14 (marker 16C) that overlaps with the melanophore QTL at marker 133B similarly shows opposite phenotypic effects (Fig. 2c). Interestingly, the exact same peak marker (214C) on linkage group 25 is associated with both reductions in eye size and more melanophores (Fig. 2d). Finally, the lens size QTL associated with NYU27 (on linkage group 6) maps near a melanophore number QTL (119C), which is associated with increased pigmentation in the homozygous cave condition (Fig. 2e).

The distances between vision and pigmentation loci varied. On linkage group 7, the ‘eye size’ locus was 28.6 cM, 38.2 cM and 51.2 cM away from the MelA, MelD and MelE loci, respectively. On linkage group 14, the ‘lens’ locus was 63 cM away from the MelA locus. On linkage group 25, the ‘lens’ locus was 3.1 cM away from the MelA locus, and on linkage group 6, the ‘lens’ locus was 9.1 cM away from the MelD locus. The distances between certain vision and melanophore number QTL (e.g., ~63 cM) were concerning, however at present we cannot rule out the possibility of falsely inflated distances between markers owing to the number genomic markers employed in this study, or whether these markers resided in a genomic interval marked by higher rates of recombination. Although a definitive determination of pleiotropy must await future analyses in which identified genes are functionally validated, we feel these distances are consistent with our hypotheses. As the current draft of the *Astyanax* genome improves, so will our ability to delimit causative loci and determine whether more than one gene mediates certain eye size and melanophore number QTL.

These results reinforce the notion that eye loss and pigmentation are highly complex, polygenic traits [10]. However, the overlap we observed between pigmentation and eye size traits revealed that both phenotypes are associated with many of the same regions of the genome (Fig. 2). Further, phenotypic effect analyses revealed that several of these genomic regions include QTL associated with eye size reduction and increased numbers of melanophores.

### Hypermelanic individuals harbor abnormal eye morphologies

Based on the results of our reanalysis of Protas et al. [10] and on other model systems (i.e., blind *Xenopus*, *eyeless* axolotls, and *lakritz* zebrafish), we predicted that hypermelanic individuals would harbor a non-functional visual system [15–17]. Spontaneously blind *Xenopus* frogs demonstrate a loss of background adaptation under ambient illumination and harbored diverse visual system malformations [15]. Thus, we presumed that analogous structural malformations and reductions in eye size present in hypermelanic hybrids were similarly accompanied by blindness.

Interestingly, a previous analysis of the *brown* phenotype provided anecdotal evidence that reduced eye size *may* impact pigmentation in certain *Astyanax* F_2_ hybrids [12]. One representative individual with dark melanization harbored a reduced eye that was sunken into the orbit of the skull (see Fig. 4a in [12]). Based on these observations, we evaluated externally-visible eye morphologies including: 1) the orbital area of the eye, 2) the eyeball area, and 3) the area of the pupillary opening of the eye. Collective values were compared across hypermelanic, surface and Pachón cavefish individuals.

**Fig. 3:**
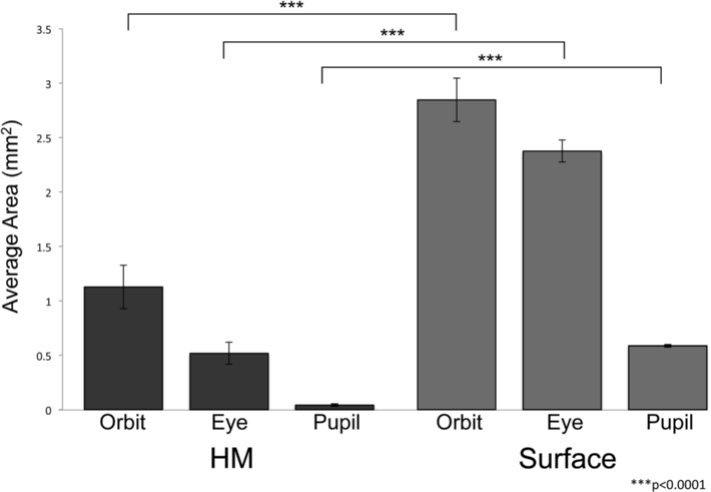
Hypermelanic fish harbor significant morphological abnormalities associated with their structural eye. Average eye size was quantified as orbital area (mm^2^), eyeball area (mm^2^), and pupil area (mm^2^) in *n* = 10 hypermelanic and *n* = 10 surface fish using the ‘polygon selection’ and ‘measurement’ tools in ImageJ. Hypermelanic fish exhibit significantly smaller eyes across all three metrics compared to surface fish (***p < 0.0001)

All hypermelanic individuals (*n* = 10) were compared against wild type surface-dwelling fish (*n* = 10; Fig. 3). Hypermelanic individuals had a mean orbital area (1.13 mm^2^) that was ~40 % of the area in surface fish (2.85 mm^2^). Measures of the mean eye area in hypermelanic fish (0.52 mm^2^) were ~22 % the size of surface-dwelling fish (2.38 mm^2^). Finally, hypermelanic fish demonstrated a ~7 % reduction in the area of the pupillary opening (0.04 mm^2^) compared to surface fish (0.59 mm^2^). These results indicate that hypermelanic fish have statistically significant reductions in quantitative measures of visual system morphologies alongside increased melanic pigmentation (Fig. 3).

For this analysis, we were unable to perform electrophysiological recordings to formally evaluate a functioning visual system. By scoring and measuring the size of visual system structures, we provide supporting evidence for the absence of vision in hypermelanic individuals. Indeed, eye size observations (and specifically “degenerative morphologies”) were key features of classic amphibian studies that first established the link between ‘blindness’ and hyperpigmentation [15]. Our results are therefore consistent with the hypothesis that hypermelanic individuals lack a functioning visual system.

### Hypermelanic fish are more similar to cavefish in a light–dark preference assay

**Fig. 4:**
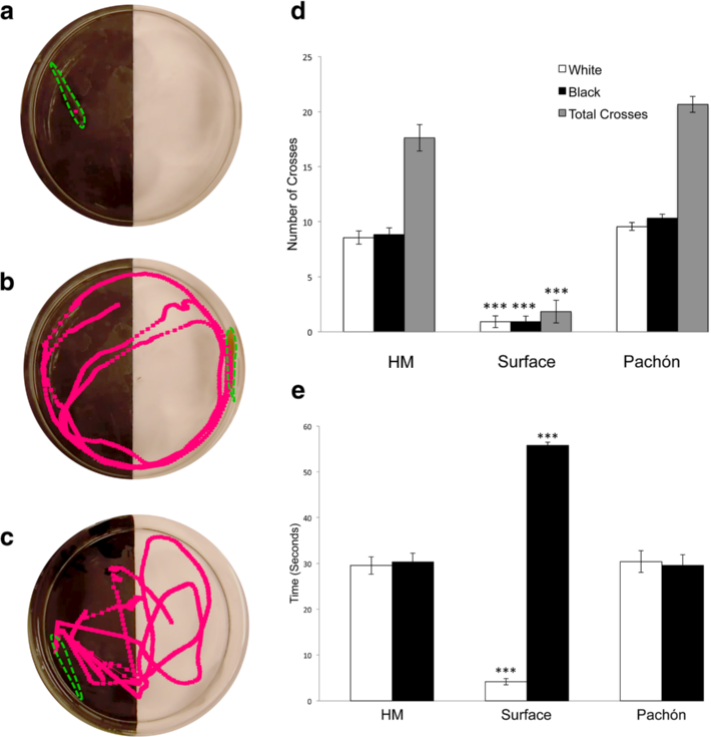
Hypermelanic fish behave similar to cavefish in a visually-mediated behavioral assay. A ‘half-moon’ arena, separated into equal white and black background halves, was used to test visual function based on predicted surface fish behavior. Representative screenshots from 1-min video trials illustrate behavior (red trace line) for individual surface fish (**a**), eyeless Pachón cavefish (**b**) and hypermelanic fish (**c**). The number of midline crosses was recorded for the total number of crosses, as well as the number of crosses into the white and black halves of the arena (**d**). Both cavefish and hypermelanic fish showed no preference to the light and dark sides of the arena, while the surface fish rarely crossed the midline. The amount of time (seconds) each fish spent on each side of the arena was also recorded (**e**). Surface fish showed a clear preference for the dark side of the arena, spending almost the entire 60 s trial in the dark, while cavefish and hypermelanic fish spent relatively equal time on both sides of the tank. For both metrics, there was no difference between hypermelanic fish and cavefish behavior, however both differed significantly from surface fish (***p < 0.0001)

If hypermelanism is associated with a nonfunctional visual system, as in other blind fish and amphibians [15–17], we reasoned that these fish would be more similar to cavefish with respect to a visual assay. To test this, we evaluated hypermelanic fish, surface, and Pachón cavefish activity in a black/white “half moon” arena (Methods; Fig. 4a-c). Surface-dwelling fish are scotophilic [22], and therefore they avoid illumination during the day as a predator-avoidance mechanism [23]. Consequently, lab-reared surface fish frequently occupy the most dimly-lit regions of a husbandry tank. Conversely, lab-reared Pachón cavefish – which lack a functional visual system – swim freely without a discernible pattern around rearing tanks.

The scotophilic response is likely mediated by vision [23, 24] and therefore a light–dark preference assay provides a simple, indirect measure of visual system function. In our assay, surface-dwelling fish (*n* = 10) remained almost exclusively in the dark section (55.78 s (s) in “black”; 4.19 s in “white”; Fig. 4a; e). This demonstrates the robust scotophilic response in surface fish during assay measurements. Conversely, eyeless Pachón cavefish spent equal time in the white (30.43 s) and the black (29.57 s) sections (Fig. 4b; e), indicating no preferential position in the half moon tank arenas. Interestingly, hypermelanic fish showed the same results as Pachón cavefish, spending roughly equal time in the white (29.56 s) and black (30.35 s) sections of the trial tanks (Fig. 4c; e).

To determine the frequency of movement within the trial tanks, we scored how frequently fish crossed the midline between white and black portions of the arena. Surface fish crossed the midline an average of 1.83 times per assay, entering into the white portion of the arena 0.9 times, and the black portion 0.9 times (Fig. 4d). Thus, surface fish spent the majority of time in the black side of the arena, usually remaining fixed in this location for the 60-s assay. In contrast, Pachón cavefish crossed the midline an average of 20.67 times during the 60 s trial period, and entered the black and white portions of the arena an average of 10.33 and 9.57 times, respectively (Fig. 4d). Similarly, hypermelanic fish crossed midline an average of 17.63 times during a 60 s trial, entering the black and white portions an average of 8.83 and 8.57 times, respectively (Fig. 4d). These results indicate hypermelanic fish are more similar to blind cave-dwelling fish based on an assay for visually-mediated activity.

We note that the light/dark assay we performed was a *proxy* measure for vision, as opposed to a more definitive measure of vision (e.g., direct electrophysiological recording of the optic nerve in response to stimulation). However, it should be noted that a wide range of physiological and behavioral dissimilarities between cave and surface fish likely influences activity differences. Here, we focused on one of the most robust and simple behavioral outputs – preference of surface fish to remain in a dark portion of a tank. We believe this is a suitable proxy for vision, since vision is required for surface fish to perform this scotophilic response. Consistent with our hypothesis, hypermelanic fish never demonstrated scotophilia or a preference for the dark portion of the assay tank.

### Dark-reared surface fish exhibit similar pigmentation levels to hypermelanic individuals

Experimentally or spontaneously blind amphibians are much darker than wild type individuals [15, 16]. However, the level of darkness exhibited by blind animals is similar to wild type animals reared on a dark background [15]. When normal vision was experimentally returned to *eyeless* axolotls, their pigmentation levels reverted to those of wild type axolotls [16]. Therefore, we reasoned that surface fish reared in darkness would exhibit darker pigmentation compared to surface fish reared under a normal 12 h light: 12 h dark photic schedule. To test this, we reared wild type surface-dwelling fish in ‘black-out’ tanks (Methods) for ~4–6 months, and quantified pigmentation levels.

We first compared melanophore numbers between hypermelanic fish, surface-dwelling fish reared under normal light/dark conditions (“S-LD”) and surface fish reared under constant darkness (“S-DD”; Fig. 1a-c). When we assessed the ventral profile region of each of the three classes of fish, we found significantly higher melanophore numbers in S-DD compared to S-LD fish (compare Fig. 1b and c). Interestingly, a similar (but not identical) pattern was observed in hypermelanic individuals (Fig. 1a). However, the partial recapitulation of hypermelanism in S-DD fish suggests that darker pigmentation is influenced by an inability to visualize ambient illumination.

A greyscale analysis revealed S-DD fish were not significantly different from S-LD individuals; and hypermelanic fish were substantially darker than both S-LD and S-DD fish (Fig. 1d). In a coloration analysis, however, S-DD fish showed substantially higher contributions of magenta and keyed channels compared to S-LD fish (Fig. 1e). Hypermelanic fish were not statistically significant from S-DD fish with respect to overall coloration (Fig. 1e). This may indicate that hypermelanic fish and S-DD are dissimilar to one another, but the nature of their pigmentation similarity is more appropriately measured through a greyscale (rather than CMYK) analysis, since our greyscale analysis revealed differences between hypermelanic and S-DD fish.

### Hypermelanic melanophores are capable of melanosome aggregation

**Fig. 5:**
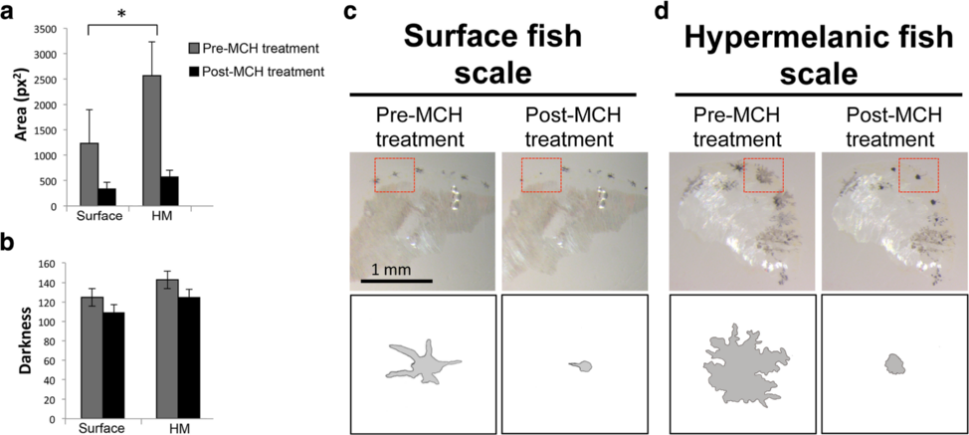
Melanophores derived from hypermelanic fish respond normally to melanin-concentrating hormone. Scale melanophores derived from hypermelanic hybrid fish and surface-dwelling fish were exposed to 1 μM of melanin-concentrating hormone (“MCH”). Both melanophore types displayed a robust response to MCH treatment, quantified as a reduction in melanophore area between untreated and MCH-treated conditions (**a**). Note that untreated hypermelanic melanophores are significantly larger than untreated surface melanophores (*p* = 0.013), however the level of darkness between melanophore types did not differ (**b**). A qualitative comparison of representative melanophore scales before and after MCH treatment reveals a robust reduction in melanophore size in response to treatment in both surface fish (**c**) and hypermelanic hybrid fish (**d**). Note that not all melanophores exhibited a response to MCH treatment. Red box in C and D depicts traced area of melanophores

Similar to blind amphibians [15, 16], melanophores in hypermelanic fish demonstrate an expanded dendritic morphology compared to surface fish reared under light–dark photic conditions (Fig. 5c; d). In principle, this morphological appearance may be rooted in a defect in the ability of hypermelanic melanophores to aggregate melanin [25]. To test this, we performed a scale assay in which we applied 1 μM of melanin-concentrating hormone to scales (Methods). This hormone, which functions as an antagonist to melanin stimulating hormone, is critical for initiating the “background adaptation” physiological response which enables organisms to lighten their skin color to blend with the surroundings [26].

Following application of MCH, hypermelanic melanophores were capable of initiating an aggregation response based on morphology of treated melanophores (Fig. 5d). The response we observed was qualitatively similar to that of scale melanophores drawn from surface-dwelling fish (Fig. 5c). This suggests that hypermelanic fish *do not* harbor a defect in their ability to normally aggregate melanophores [27].

Interestingly, the overall area of untreated hypermelanic fish melanophores was substantially larger than those collected from surface fish (Fig. 5a). Following MCH treatment, however, the morphology of melanophores from both hypermelanic and surface fish were similar (Fig. 5c; d). When we measured darkness of melanophores, we did not observe significant differences between hypermelanic and surface fish (Fig. 5b). Overall, our results indicate that hypermelanic fish maintain the ability to aggregate melanin within melanophores, and that larger melanophores are a feature of the hypermelanic phenotype.

## Discussion

### *Vision loss and hypermelanism are linked in certain hybrid* Astyanax *pedigree members*

Hypermelanic fish were qualitatively more similar to surface-dwelling fish with respect to pigmentation. However, using a proxy measure for vision, hypermelanic fish appeared to be blind based on similarity of behavior with cavefish in a light–dark swimming assay. We predicted if reduced vision can impact pigmentation in F_2_ individuals, certain genomic regions would be associated with reduced vision and increased pigmentation. In a reanalysis of the work of Protas et al. [10], we found eleven genomic regions shared between both traits. Four of these genomic regions demonstrated phenotypic polarities wherein homozygous cave conditions were associated with reduced eye size and increased melanophore numbers. These results are consistent with the notion that darker pigmentation in hybrids can arise, at least in part, from aberrant vision.

Several classical studies similarly demonstrated this indirect relationship using blinded amphibians harboring darker pigmentation [28–30]. More recent studies have shown that this phenomenon is also present in fish [17]. Our study demonstrates that certain hybrid individuals bred from a Pachón cavefish x surface fish cross demonstrate melanic pigmentation exceeding that of surface fish reared under normal conditions. Hybrid hypermelanic fish are most likely functionally blind, since every individual harbored severe morphological aberrations of the eye (Fig. 1a), and were indifferent in a visual behavioral assay (Fig. 4c). In these individuals, lack of visual input likely causes darkened pigmentation via a pleiotropic effect of vision loss on melanization.

This phenomenon has been described in *Xenopus* frogs [15], *eyeless* axolotls [16], and *lakritz* mutant zebrafish [17]. In all three systems, blind forms are substantially darker than wild type individuals. Interestingly, when vision was experimentally restored to *eyeless* axolotls through optic cup transplantations, normal pigmentation returned [16]. Moreover, the defect was not rooted in abnormal neural crest tissues (i.e., the embryonic tissue of origin for melanophores), since transplanted explants of the cranial neural crest between *eyeless* and wild type individuals had no impact on melanic phenotype. Thus, the key phenotype associated with hypermelanism in *eyeless* axolotls was absence of vision [16].

We found several parallels between hypermelanic hybrid *Astyanax* fish and *eyeless* axolotl mutants. First, overall melanophore morphology and appearance in hypermelanic individuals were similar to other reports. Specifically, melanophores were more stellate in shape, larger, and were increased in numbers [15–17]. Additionally, as shown in other model systems [15, 16], hypermelanic fish demonstrate distribution patterns distinct from those found in normal surface-dwelling fish, particularly in the ventral region of the head (Fig. 1a, asterisk), as well as numerical increases in melanophores (see melanophore counts; [10]). The darker pigmentation observed in blind *Xenopus* frogs, axolotls, and zebrafish resulted from a pleiotropic consequence of vision loss [15–17]. Consistent with this, every hypermelanic individual we observed by direct observation harbored significant morphological defects in the visual system.

### Hypermelanism in hybrids is mediated by vision loss rather than a specific genotype

Our reanalysis of the pedigree reported in Protas et al. [10] demonstrated that hypermelanism can have diverse genetic underpinnings and be manifested in different ways. For example, from the eleven putatively hypermelanic individuals we identified from the pedigree studied in Protas et al. [10], no consistent genotypes were associated with hypermelanism (Additional file 1: Table S1). Thus, the darker pigmentation expressed in certain hybrid fish is not a simple trait governed by one or a few loci, but rather a general phenomenon most likely arising principally through vision loss.

Several genes are associated with pigmentation loss in *Astyanax*, and any of a number of combinatorial genotypes can be present within experimental F_2_ hybrids [18]. This may explain, in part, the (very few) number of hybrid individuals expressing the hypermelanic phenotype. For instance, in order to express hypermelanism, a given hybrid fish could not also express albinism as it would mask the normal expression of any melanin. The precise number and identity of pigmentation genes that must be present in order to express this trait, however, remains unknown.

Similarly, the genes crucial for conferring functional blindness in hybrids remain unknown. However, presumably there are multiple genes [10] that can be inherited in a combinatorial fashion and result in blindness in *Astyanax* hybrids. As with pigmentation, eye loss in *Astyanax* cavefish is polygenic trait [18, 31, 32]. The identity and role of particular ‘eye loss’ loci in the expression of hypermelanism awaits future studies identifying the genes that underlie these QTL.

We believe that blindness is the most likely feature contributing to the loss of a normal skin lightening response. As shown in prior studies [15–17, 28–30], blind fish are unable to perform visually-controlled background adaptation under ambient illumination, and as a consequence always appear darker than wild type animals. As demonstrated in prior studies, the pigmentation phenotype is a secondary consequence of visual system disorders. In light of studies in *Astyanax* demonstrating diverse QTL polarities associated with melanophore numbers, it will be crucial to determine which of the genes underlying these QTL can demonstrate differential effects in the context of a blinded individual.

### Hypermelanic hybrid fish demonstrate pigmentation phenotypes similar to other blinded fish and amphibians

For over 80 years, it has been appreciated that removal of the eyeball can induce a change in melanin granules leading to a permanent, darker phenotype [28]. This phenotype was demonstrated in a variety of experimentally or spontaneously blind model systems, including *Xenopus* and *Rana* frogs [15, 28–30], salamanders [16] and fish [17]. Classic studies revealed that hypothalamic lesions yielded the same ‘darkening’ phenotypic effect [33, 34], suggesting that continuous release of melanin-stimulating hormone (MSH) from the pars intermedia of the pituitary gland mediates the hypermelanic phenotype. Interestingly, a gene mutated in several cave populations, *Mc1r*, encodes the receptor that binds MSH and augments melanic pigmentation [8, 12].

A number of pigment phenotypes underlie this darkened appearance, including increased size and branching of melanophores, increase in melanophore number, and altered melanophore distribution – especially to ventral regions of the animal not normally occupied by pigment cells. In other systems, wild type individuals approached a comparable level of darkness through rearing on a darkened background [15]. Here, we demonstrated that hypermelanic hybrid *Astyanax* demonstrate the same characteristics of increased melanophore sizes (Fig. 5a) and branching (Fig. 5c; d). Our reevaluation of the work of Protas et al. [10], as well as a second experimental F_2_ pedigree, revealed hypermelanic individuals have more melanophores and the presence of melanophores in ventral regions (Fig. 1a). In addition, dark-reared wild type individuals approach the same level of darkness as hypermelanic fish, as in previous studies [15]. Thus, the external ‘darkened’ phenotype demonstrated by hypermelanic *Astyanax* hybrids closely resembles that of experimental and naturally blinded animals.

In sum, this work indicates that *Astyanax mexicanus* and experimental F_2_ hybrids demonstrate the same loss of background adaptation as a consequence of vision loss, as shown in other fish and amphibians. Experimental hybrid individuals revealed this pleiotropic interaction between vision and pigmentation. It is unclear if this phenomenon influenced QTL polarity studies owing to the small number of individuals demonstrating hypermelanism. However, it is intriguing that this phenomenon persists in cave-dwelling *Astyanax* given the absence of vision in many ‘pure-bred’ cave populations. Indeed, increased pigmentation resulting from vision loss may incur an energetic cost for organisms living in complete darkness. This may explain, in part, why pigmentation is repeatedly lost in obligate cave-dwelling organisms. If vision loss imposes an energy cost (through enhanced melanization), pigmentation may be *negatively selected* against to minimize this energy drain in the nutrient poor environment of the cave. In sum, this work indicates that genetic and phenotypic studies in *Astyanax* will benefit from integrative analyses that rely on both direct scoring of quantitative traits, as well as potential interactions between traits.

### An alternative explanation for the interaction between pigmentation and vision

Based on the precedent in axolotls and frogs, we assume that hypermelanism results from lack of vision. However, an interesting alternative is that hyperpigmentation may negatively impact vision. A direct relationship between pigmentation condition, and specifically albinism, on multiple aspects of vision has long been appreciated. For instance, individuals with albinism demonstrate reduced visual resolution and acuity [35], optic nerve misrouting [36], foveal hypoplasia [37], and reduced retinal cell numbers [38]. These processes are underpinned by multiple effects from the retinal pigmented epithelium [39], which is crucial for normal retinal development [40]. If loss of pigmentation, can negatively impact vision – can hyperpigmentation have similar negative effects?

Interestingly, many clinical cases of macular degeneration are marked by hyperpigmentation of the retinal pigment epithelium [41]. Further, a previous study noted that important predictors for progression of age-related macular degeneration included retinal pigmentary changes (inclusive of both depigmentation and hyperpigmentation; [42]). Areas of hyperpigmentation in the retinal pigment epithelium are associated with pathogenic tears that are unable to support photoreceptor cells, leading to reduced vision [43]. Therefore, it is possible that hypermelanism, and specifically hyperpigmentation of the retinal pigment epithelium, could negatively impact vision in hypermelanic individuals. Given the fact that dark-reared surface fish in our study did not produce a significant increase in pigmentation may suggest that a direct link between hypermelanism and vision cannot be ruled out. Moreover, it may be that these traits can simultaneously affect one another – reduced vision may enhance pigmentation, and enhanced pigmentation may lead to visual system defects. Additional studies are necessary to parse out the relative influence of hypermelanism and vision loss on one another.

### Using experimental versus natural hybrid fish to evaluate hypermelanism

This study focused on the use of *experimental hybrids* (non-natural individuals, produced in captivity) as a means of evaluating meiotic events and the recombination of “parental” alleles (surface and cavefish). Experimental hybrid individuals, alongside QTL approaches, can enable discovery of genomic regions associated with trait inheritance and identify candidate genes that may underlie a trait. However, “natural” hybrids, can also be found in the wild, particularly at certain caves (e.g., Chica) and surface-dwelling forms (for a comprehensive review, see [44]). Specifically at the Pachón cave locality, recent population genetic studies provide evidence of introgression between cave and surface populations, followed by periodic migration events, throughout the geologic past [45–47]. Unlike the experimental hybrids we evaluated here, natural hybrids may provide the opportunity to test the assumption that natural hybrids experience increased melanization alongside reduced eye sizes. The precise energetic “cost” of hyperpigmentation is unknown, however if natural hypermelanic hybrid fish are not found in wild, it may be that negative selection acts on individuals demonstrating hyperpigmentation.

An important consideration, however, is that the condition observed in extant hybrids between cave and surface-dwelling forms likely does not recapitulate the ancestral condition for either parental morphotype. This is because extant cave and surface populations harbor their own respective evolutionary lineages, and therefore their genetic constitution likely differs from earlier incipient cave-dwelling forms [44]. Experimental evidence to support this notion comes from a recent paper examining the energetic costs of vision [48]. In this study, the authors observed a close correlation between eye and optic tectum sizes in Pachón cavefish, surface fish and Micos cavefish (a “younger” cave lineage with an intermediate level of eye regression). This works suggests the eye and the optic tectum remained anatomically “coupled” throughout evolution. When this anatomical relationship was evaluated in experimental hybrid fish, the correlation between these structures disappeared. Thus, phenotypes evident in contemporary cave x surface natural hybrids (whether natural or experimental) cannot necessarily be used as a surrogate for the ‘ancestral’ form of either parental morphotype.

### The energetic cost of pigmentation

The hyperpigmentation phenomenon we describe in this report may impose an energetic cost. In birds, there is evidence of an energetic cost to carotenoid utilization in generating colorful plumage patterns [49]. However, similar measures of precise “costs” of melanization in surface and cave forms of *Astyanax* are incompletely understood. Such an energetic liability could conceivably occur during early development, throughout adulthood to maintain pigmentation, or across several stages of life history. Without further analysis, it is impossible for us to know the strength of these putative “costs” for pigmentation. However, in most eyeless populations, including Pachón cavefish, an eye develops early in development, but subsequently degrades, sinks into the orbit and is degenerated by adulthood [2]. This may indicate differential pressures to lose vision across life history. As an embryo, the developmental costs are evidently not overwhelming, since the eye begins to form. However, as an adult, perhaps owing to the energetic demands of visual system maintenance, the eye degenerates. It is intriguing to think that a similar progression may also occur with melanization. However, dynamic changes in cavefish pigmentation (and specifically, changes in melanophore numbers over life history) to our knowledge have not yet been examined.

An important consideration concerning energetic costs is that “pigmentation” is a highly complex, multifactorial trait. Pigmentation in *Astyanax* is governed by three different cell types (melanophores, xanthophores, and iridophores), and composed of both simple Mendelian traits (e.g., albinism, *brown*) and complex, polygenic traits (e.g., melanophore number variation; [12]). Additionally, each of these traits (with the exception of albinism) is also likely vulnerable to environmental influences. Pigmentation is a complex phenotype [8] and several equally complex forces likely govern its loss in cave animals. Indeed, selective, neutral, or a combination of both forces may explain different components of pigmentation loss. This study focused on ‘melanophore number’ variation. Selective pressures to reduce melanophore numbers may be present during the initial stages of evolutionary regression of pigmentation, in which case hypermelanic individuals would likely experience negative selection to conserve energy in the nutrient-poor cave microenvironment.

### Hypermelanism and QTL “clustering” in troglomorphic evolution

The work we present here provides support for the notion of “clustering” of QTL associated with troglomorphic changes. These clusters have long been appreciated in *Astyanax*, stretching from the first linkage studies [50], through a recent comprehensive review of *Astyanax* QTL analyses [51]. Many studies have found substantial co-localization of significant loci linked to a variety of different phenotypes. These traits have included a number of “regressive” and “constructive” phenotypes [10, 18], numerical variation in sensory neuromasts [52], and a variety of craniofacial measures [21]. The proximity of multiple QTL in the same genomic regions may provide a quick route towards evolving troglomorphic features that are adaptive within the darkness of a cave [53]. The phenomenon of hypermelanism documented here provides support for this notion since multiple different traits are united through one or a few QTL. Although the QTL we discuss were previously discovered and documented by Protas et al. [10], our observation of hypermelanism as arising indirectly through vision loss ties seemingly disparate phenotypes together through the same, or closely linked, loci.

## Conclusions

The ability to blend into surroundings (background adaptation) is a common feature among fish and amphibians [16, 17]. The work we present here, based on hybrid individuals, revealed that this physiological process was likely retained in early cave-dwelling *Astyanax* populations*.* Background adaptation is somewhat paradoxical in cave-dwelling fish since it hinges on vision. The visual system is regarded as having been lost under selective pressure, evidenced by phenotypic effect studies [10]. In other cave-dwelling animals, vision is believed to have been lost earlier than other regressive phenotypes, including pigmentation [4]. However, as vision regressed in early cave-dwelling lineages, the result was constitutive release of MSH from the pituitary gland, yielding an overproduction of melanin [15]. Thus, the evolutionary loss of vision was not without consequence. Through a pleiotropic interaction, enhanced pigmentation resulted in the early cave colonizers. This is counterintuitive since the most conspicuous traits absent from extant cave-dwelling lineages are both vision *and* pigmentation [5]. We propose that the pleiotropic interaction between vision loss and enhanced pigmentation led to an energetic cost in the nutrient-poor environment of the cave. This energetic cost was probably selected against, as a means of energy conservation [2]. This negative selection against enhanced melanin production likely contributed to the phenotypic regression of pigmentation evident in extant cave-dwelling fish. Indeed, two Mendelian pigment phenotypes (albinism and *brown*) are both widespread, highly penetrant, and mediated through loss-of-function mutations in the genes *Oca2* and *Mc1r*, respectively [12, 54]. Recent studies have provided evidence of selection for albinism as a means of increasing catecholamine levels, which promote adaptive feeding and sleep behaviors in *Astyanax* cavefish [7]. Therefore, the “interaction” we report here between decreased vision and increased pigmentation reveals an additional selective mechanism through which pigmentation was lost in cave-dwelling animals.

## Abbreviations

μm, micrometer; CMYK, “cyan, magenta, yellow, keyed”; d, day; DD, “dark/dark”, specifically a rearing photic schedule of 24 h of darkness; F_2_, a second-generation experimental pedigree; hr, hour; LD, “light/dark”, specifically a rearing photic schedule of 12 h of light, 12 h of darkness; mm, millimeter; QTL, “quantitative trait locus”.

## Additional file

Additional file 1: Table S1. Genotypes of hybrid F_2_ individuals demonstrating dark pigmentation and small eyes (data reanalyzed from Protas et al. 2007). (XLSX 10 kb)

## References

[1] Barr TC (1968). Cave ecology and the evolution of troglobites. Evol Biol.

[2] Jeffery WR (2001). Cavefish as a model system in evolutionary developmental biology. Dev Biol.

[3] Yamamoto Y, Stock DW, Jeffery WR (2004). Hedgehog signalling controls eye degeneration in blind cavefish. Nature.

[4] Klaus S, Mendoza JCE, Liew JH, Plath M, Meier R, Yeo DCJ (2013). Rapid evolution of troglomorphic characters suggests selection rather than neutral mutation as a driver of eye reduction in cave crabs. Biol Lett.

[5] Gross JB (2012). The complex origin of *Astyanax* cavefish. BMC Evol Biol.

[6] McGaugh SE, Gross JB, Aken B, Blin M, Borowsky R (2014). The cavefish genome reveals candidate genes for eye loss. Nat Comm.

[7] Bilandzija H, Ma L, Parkhurst A, Jeffery WR (2013). A potential benefit of albinism in *Astyanax* cavefish: downregulation of the *Oca2* gene increases tyrosine and catecholamine levels as an alternative to melanin synthesis. PLoS One.

[8] Stahl BA, Gross JB (2015). Alterations in *Mc1r* gene expression are associated with regressive pigmentation in *Astyanax* cavefish. Dev Genes Evol.

[9] Wilkens H. Beiträge zur Degeneration des Melaninpigments bei cavernocolen Sippen des *Astyanax mexicanus* (Filippi). Z Zoot Syst Evolutionsforsch 1970a;8:173–199.

[10] Protas M, Conrad M, Gross JB, Tabin C, Borowsky R (2007). Regressive evolution in the Mexican cave tetra, *Astyanax mexicanus*. Curr Biol.

[11] Wilkens H (1971). Genetic interpretation of regressive evolutionary processes: studies on hybrid eyes of two *Astyanax populations* (Characidae, Pisces). Evolution.

[12] Gross JB, Borowsky R, Tabin CJ (2009). A novel role for *Mc1r* in the parallel evolution of depigmentation in independent populations of the cavefish *Astyanax mexicanus*. PLoS Genet.

[13] Wilkens H. Beiträge zur Degeneration des Auges bei Cavernicolen, Genzahl und Manifestationsart: Untersuchungen an mexikanischen Höhlenfischen. J Zool Syst Evol Res 1970b;8: 1–47.

[14] Wilkens H (1976). Genotypic and phenotypic variability in cave animals. Studies on a phylogenetically young cave population of *Astyanax mexicanus* (Fillippi). Ann Spéléol.

[15] Imai K, Takahashi H (1971). Changes in the melanophorotropic function of the pituitary gland accompanying blindness in *Xenopus laevis* Daudin. Dev Growth Differ.

[16] Epp LG (1972). Development of pigmentation in the eyeless mutant of the Mexican axolotl, *Ambystoma mexicanum*, shaw. J Exp Zool.

[17] Kay JN, Finger-Baier KC, Roeser T, Staub W, Baier H (2001). Retinal ganglion cell genesis requires *lakritz*, a zebrafish *atonal* homolog. Neuron.

[18] Protas M, Tabansky I, Conrad M, Gross JB, Vidal O, Tabin CJ, Borowsky R (2008). Multi-trait evolution in a cave fish, *Astyanax mexicanus*. Evol Dev.

[19] Şadoğlu P, McKee A (1969). A second gene that affects eye and body color in Mexican blind cave fish. J Hered.

[20] Gross JB, Wilkens H (2013). Albinism in phylogenetically and geographically distinct populations of *Astyanax* cavefish arises through the same loss-of-function *Oca2* allele. Heredity.

[21] Gross JB, Krutzler AJ, Carlson BM (2014). Complex craniofacial changes in blind cave-dwelling fish are mediated by genetically symmetric and asymmetric loci. Genetics.

[22] Romero A (1985). Ontogenetic change in phototactic responses of surface and cave populations of *Astyanax fasciatus* (Pisces: Characidae). Copeia.

[23] Stewart A, Maximino C, de Brito TM, Herculano AM, Gouveia Jr A, Morato S, Cachat JM, Gaikwad S, Elegante MF, Hart PC, Kalueff AV. Neurophenotyping of adult zebrafish using the light/dark box paradigm. In: Zebrafish Neurobehavioral Protocols, Humana Press; 2011. pp. 157–167.

[24] Romero A, Green SM, Romero A, Lelonek MM, Stropnicky KC (2003). One eye but no vision: cave fish with induced eyes do not respond to light. J Exp Zool B Mol Dev Evol.

[25] Oshima N, Nakamaru N, Araki S, Sugimoto M (2001). Comparative analyses of the pigment-aggregating and -dispersing actions of MCH on fish chromatophores. Comp Biochem Physiol C: Toxicol Pharmacol.

[26] Rance T, Baker BI (1979). The teleost melanin-concentrating hormone: a pituitary hormone of hypothalamic origin. Gen Comp Endocrinol.

[27] Logan DW, Burn SF, Jackson IJ (2006). Regulation of pigmentation in zebrafish melanophores. Pigment Cell Res.

[28] Hogben L, Slome D (1931). The pigmentary effector system. VI. The dual character of endocrine coordination in amphibian colour change. Proc R Soc Lond B Biol Sci.

[29] Rowlands A (1952). The influence of water and light upon the colour change of sightless frogs (*Rana temporaria*). J Exp Biol.

[30] Charlton HM (1966). The pineal gland and color change in *Xenopus laevis* Daudin. Gen Comp Endocrinol.

[31] Wilkens H, Hecht MK, Wallace B (1988). Evolution and genetics of epigean and cave *Astyanax fasciatus* (Characidae, Pisces): support for the neutral mutation theory. Evolutionary biology.

[32] Wilkens H, Strecker U (2003). Convergent evolution of the cavefish *Astyanax* (Characidae: teleostei): genetic evidence from reduced eye-size and pigmentation. Biol J Linnean Soc.

[33] Kastin A, Ross GT (1965). Melanocyte-stimulating hormone activity in pituitaries of frogs with hypothalamic lesions. Endocrinology.

[34] Ito T (1968). Experimental studies on the hypothalamic control of the pars intermedia activity of the frog, *Rana nigromaculata*. Neuroendocrinology.

[35] Abadi RV, Pascal E (1991). Visual resolution limits in human albinism. Vision Res.

[36] Creel DJ, Summers CG, King RA (1990). Visual anomalies associated with albinism. Ophthalmic Paediatr Genet.

[37] McAllister JT, Dubis AM, Tait DM, Ostler S, Rha J, Stepien KE, Summers CG, Carroll J (2010). Arrested development: high-resolution imaging of foveal morphology in albinism. Vision Res.

[38] Hagen EA, Houston GC, Hoffmann MB, Jeffery G, Morland AB (2005). Retinal abnormalities in human albinism translate into a reduction of grey matter in the occipital cortex. Eur J Neurosci.

[39] Katz ML, Parker KR, Handelman GJ, Bramel TL, Dratz EA (1982). Effects of antioxidant nutrient deficiency on the retina and retinal pigment epithelium of albino rats: a light and electron microscopic study. Exp Eye Res.

[40] Raymond SM, Jackson IJ (1995). The retinal pigmented epithelium is required for development and maintenance of the mouse neural retina. Curr Biol.

[41] Wang JJ, Foran S, Smith W, Mitchell P (2003). Risk of age-related macular degeneration in eyes with macular drusen or hyperpigmentation: the Blue Mountains Eye Study cohort. Arch Ophthalmol.

[42] Klaver CC, Assink JJ, van Leeuwen R, Wolfs RC, Vingerling JR, Stijnen T, Hofman A, de Jong PT (2001). Incidence and progression rates of age-related maculopathy: the Rotterdam study. Invest Ophthalmol Vis Sci.

[43] Meyer CH, Toth CA (2001). Retinal pigment epithelial tear with vitreomacular attachment: a novel pathogenic feature. Graefes Arch Clin Exp Ophthalmol.

[44] Wilkens H. Genetics and hybridization in surface and cave *Astyanax* (Teleostei): A comparison of regressive and constructive traits. Biol J Linnean Soc 2016; In press.

[45] Hausdorf B, Wilkens H, Strecker U (2011). Population genetic patterns revealed by microsatellite data challenge the mitochondrial DNA based taxonomy of *Astyanax* in Mexico (Characidae, Teleostei). Mol Phylogenet Evol.

[46] Strecker U, Hausdorf B, Wilkens H (2012). Parallel speciation in *Astyanax* cave fish (Teleostei) in Northern Mexico. Mol Phylogenet Evol.

[47] Coghill LM, Hulsey CD, Chaves-Campos J, de Leon FJ, Johnson SG (2014). Next generation phylogeography of cave and surface *Astyanax mexicanus*. Mol Phylogenet Evol.

[48] Moran D, Softley R, Warrant EJ (2015). The energetic cost of vision and the evolution of eyeless Mexican cavefish. Sci Adv.

[49] Hill GE (2000). Energetic constraints on expression of carotenoid-based plumage coloration. J Avian Biol.

[50] Borowsky R, Wilkens H (2002). Mapping a cave fish genome: polygenic systems and regressive evolution. J Hered.

[51] O’Quin K, McGaugh SE. Mapping the genetic basis of troglomorphy in *Astyanax*: How far we have come and where do we go from here? In: Biology and Evolution of the Mexican Cavefish, Elsevier Press; 2016. pp. 111–135.

[52] Yoshizawa M, O’Quin KE, Jeffery WR (2012). Evolution of an adaptive behavior and its sensory receptors promotes eye regression in blind cavefish. BMC Biol.

[53] Yoshizawa M, O’Quin KE, Jeffery WR (2013). QTL clustering as a mechanism for rapid multi-trait evolution. Commun Integr Biol.

[54] Protas ME, Hersey C, Kochanek D, Zhou Y, Wilkens H, Jeffery WR, Zon LI, Borowsky R, Tabin CJ (2006). Genetic analysis of cavefish reveals molecular convergence in the evolution of albinism. Nat Genet.

[55] Gross JB, Powers AK, Davis EM, Kaplan SA (2016). Data from: a pleiotropic interaction between vision loss and hypermelanism in *Astyanax mexicanus* cave x surface hybrids.

